# Association between hearing loss level and degree of discomfort introduced by tinnitus in workers exposed to noise

**DOI:** 10.1016/S1808-8694(15)30148-8

**Published:** 2015-10-18

**Authors:** Adriano Dias, Ricardo Cordeiro

**Affiliations:** a PhD, Technical Coordinatora; b Associate Professor. Grupo de Apoio à Pesquisa/Faculdade de Medicina de Botucatu/UNESP

**Keywords:** occupational noise, noise-induced hearing loss, tinnitus

## Abstract

Hearing loss and tinnitus impact the lives of workers in every instance of their lives. **Aim**: this paper aims to investigate the existence of a dose-response relationship between hearing loss and tinnitus by determining whether higher levels of hearing loss can be associated with increased tinnitus-related discomfort. **Materials and method**: this cross-sectional case study assessed 284 workers exposed to occupational noise through pure tone audiometry. Test results were categorized as defined by Merluzzi. Individuals complaining of tinnitus answered the adapted and validated Brazilian Portuguese version of the Tinnitus Handicap Inventory. A generalized linear model was adjusted for binomial data to test the interaction between these factors. **Results**: over 60% of the ears analyzed had hearing loss, while more than 46% of them had tinnitus. Tinnitus prevalence and risk rates increased as pure tone audiometry results got worse. The association between both, considering all hearing loss degrees, was statistically significant. **Conclusion**: the results point to a statistical association between hearing loss and tinnitus; the greater the hearing loss, the greater the discomfort introduced by tinnitus.

## INTRODUCTION

Noise is the physical noxious agent most commonly found in the work environment^1^, ^2^, ^3^. The World Health Organization estimates that approximately 15% of the workers in developed countries are exposed to noise levels which are harmful to hearing^4^.

Hearing losses, being caused by occupational exposure to noise (such as those which are noise-induced) or by another agent and its effects, are among the major difficulties faced by those affected, here we discuss the workers. Among the effects accruing from hearing loss, we stress tinnitus, which besides causing difficulties in the work environment also has a negative impact on the quality of life of the worker and the people around him/her.

Tinnitus is defined as “an auditory illusion, that is, the sensation of a sound that is not associated with an external source of stimulation”^5^ or as “an occurrence in the absence of vibratory or mechanical activity corresponding to the middle or inner ear “^6^, meaning that tinnitus is a ghost auditory perception, perceived only by the affected person in most cases, and this fact makes it very difficult to measure it. Tinnitus can be seen in different ways and the objective findings as to its measures are rather controversial. For these reasons, no form of reliable measurement has been incorporated to the audiologic routine. The consensus currently accepted is that the tinnitus sound frequency is near the frequency and intensity of the hearing loss or the highest level of the hearing loss. Still, in relation to measuring tinnitus, it is not considered the most severe only because it is perceived in high intensity^7^ - since more than 80% of the complaining individuals perceive it at less than 20dB (equivalent to a whisper), while less than 5% report it to be higher than 40dB^8^. The other factors associated with a worsening of the tinnitus are: the sound type, its consistency and location^9^.

Since there is no reliable objective measurement, or one with clinical usefulness, for that matter, one of the ways to assess tinnitus is to ask the affected individual to describe the sound he/she perceives. In most of the cases it is associated to some external source of noise - such as insects, whistle or background noise, explosions, running water noise, radio or TV out of tune, wind, paper being crushed, humming and also some combination of them - the so called multiple tinnitus, which is rare. About 25% of patients report tinnitus as a pure tone^8^.

The large individual variability and the level of interference in the daily lives of people, coupled to the fact that objective testing bring about very little useful information as to the severity of tinnitus^10^, ^11^, there is no relationship between the perception of tinnitus intensity and the complaint of the disability caused by it^12^ and the sound description does not allow for a clarification of the cases. These facts have all motivated a new focus on the investigations: the assessment of tinnitus consequences through questionnaires that could quantify the psycho-emotional and functional impairments caused by it, aiming at universalizing the criteria and comparisons among populations.

**Figure 1:**
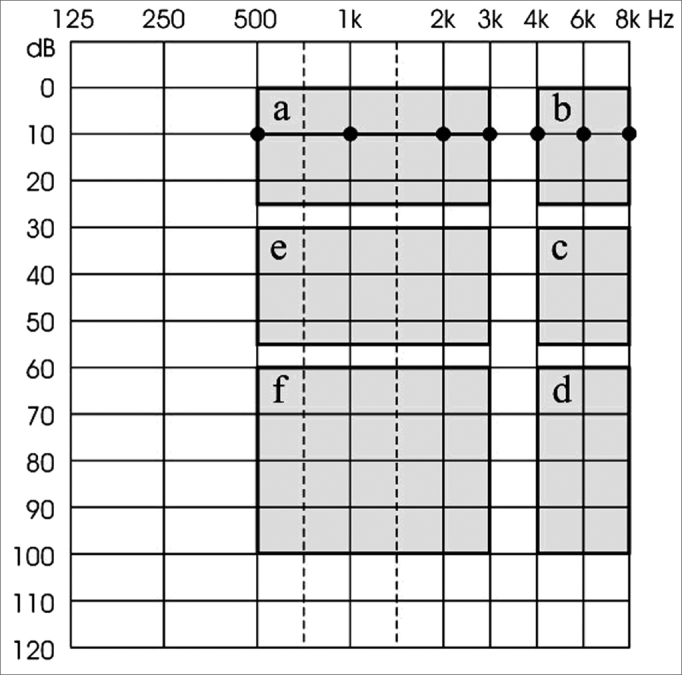
Noise-induced hearing loss classification criteria suggested by Merluzzi et al.(1979).

In order to quantify the hearing loss in the present investigation, we chose an occupational criteria (because the series is made up of workers with prior history of exposure to noise), the one suggested by Merluzzi et al.^13^, that divides the audiogram area in six sectors and allows for a distribution and characterization of the audiometric results in eight possible configurations or levels of hearing loss according to the description showed in Figure 1.

Merluzzi”s Group 0 (or normal) gathers together all the audiograms with normal curves, in other words, hearing thresholds equal to or below 25dB for all the frequencies tested (thresholds in area A). Group 1 gathers the audiograms with unaffected thresholds between 500Hz and 3kHz in area A and the thresholds between 4 and/or 6kHz are below 25dB, in other words, in areas C or D (the really important frequencies for our “social hearing” are OK: 500Hz, 1, 2 and 3kHz); Group 2 audiograms have 500Hz, 1 and 2kHz frequencies in thresholds confined to area A; 3kHz must be in areas E or F and from 4 to 8kHz may be in the remaining areas (B, C, or D); Group 3 audiograms had 500Hz and 1kHz frequencies with thresholds in area A, 2kHz and 3kHz in areas E or F and from 4 to 8kHz that may be in the remaining areas (B, C or D); Group 4 audiograms had frequency 500Hz with thresholds in area A, 1 to 3kHz in areas E or F and 8kHz that can be in the remaining areas (B, C or D); and Group 5 audiograms which are not in area A, in other words, all the thresholds are low, added to the situation that the high frequencies must be more affected than the middle and low frequencies. Group 6 is made up of all the audiometric curves which suggest the presence of two or more pathologic agents, and one of them must be noise; while Group 7 has audiograms with other hearing loss types not allegedly associated with noise.

In order to assess tinnitus, we chose the Tinnitus Severity Questionnaire (TSQ) - Questionário de Gravidade do Zumbido (QGZ)14, which is the Brazilian version of the Tinnitus Handicap Inventory - THI15, which use is justified by the fact that it has excellent validity and high internal consistency^16^, ^17^, besides being easy and quick to use (it takes about five minutes) and reproduce (some questionnaires have copyrights, but not this one). Some references corroborate the maintenance of an internal consistency of this tool even after translations and adaptations to other populations and languages^11^, ^14^, ^18^. TSQ is made up of twenty five questions, broken down in three groups. The first considers the functional component (F) of the mental level impairment (difficulty to concentrate or read), physical, social and work impairment (hearing impairment). The Emotional Group (E) measures the affective responses such as frustration, stress and depression. The last group, the so called catastrophic (C), aims at quantifying the hopelessness and the disability reported by the patient affected by tinnitus. There are three response options for each one of the questions, scored as follows: 4 points for YES, 2 points for SOMETIMES and zero for NO^15^.

Adding the resulting score of the questions, it goes from 0 (zero or 0% - all answers are NO) - when tinnitus does not impact the patient”s life, all the way to 100 (one hundred points, or 100% - all the answers are YES) - when the level of impairment is maximum, it can be classified in five groups or levels of severity. According to the classification proposed by McCombe et al.^16^ (2001), tinnitus can be: negligible (0-16%), mild (18-36%), moderate (38-56%), severe (58-76%) or catastrophic (78-100%). The 25 questions and pertaining scales which make up the TSQ are presented on Table 1.

**Table 1:** Tinnitus Severity Questionnaire (TSQ)^14^, with the respective classification scales.

1F	Do you have difficulties concentrating because of your tinnitus?
2F	Do you have difficulties hearing people because of how loud your tinnitus is?
3E	Does your tinnitus make you angry?
4F	Does your tinnitus make you confused?
5C	Do you feel hopeless because of your tinnitus?
6E	Do you complain much of your tinnitus?
7F	Do you have trouble sleeping at night because of your tinnitus?
8C	Do you feel you can not get rid of your tinnitus?
9F	Does your tinnitus interfere with your capacity to appreciate social activities (such as having dinner in a restaurant or going to the movies)?
10E	Do you feel frustrated because of your tinnitus?
11C	Because of your tinnitus, do you feel you have a terrible disease?
12F	Does your tinnitus make it difficult for you to appreciate life?
13F	Does your tinnitus impact your work or your other home chores?
14E	Do you feel you are constantly annoyed by your tinnitus?
15F	Do you have difficulties reading because of your tinnitus?
16E	Does your tinnitus make you feel bad?
17E	Do you feel your problem with tinnitus has stressed your relationship with your family and friends?
18F	Do you find it difficult to pay attention to things which are not your tinnitus?
19C	Do you feel you can not control your tinnitus?
20F	Do you frequently feel tired because of your tinnitus?
21E	Do you feel depressed because of your tinnitus?
22E	Do you feel stressed because of your tinnitus?
23C	Do you feel you can no longer deal with your tinnitus?
24F	Does your tinnitus get worse when you are stressed?
25E	Does your tinnitus make you feel insecure?

Thus, the goal of the present investigation is to check for the existence of a dose-response relationship between hearing loss and tinnitus, that is, if a worsening in audiometric thresholds is associated with the discomfort caused by tinnitus.

## MATERIALS AND METHODS

For this cross-sectional study, approved by the institution”s Ethics Committee, under protocol # 099/2002, the information collection and audiometric testing were carried out in two audiometry wards located in the city of Bauru, in Southeastern Brazil. 284 workers with prior history of exposure to occupational noise between April and October of 2003 were interviewed. The age of the workers varied between 20 and 72 years, with mean age of 42.05 (±12.49) years and median of 42 years. Distribution by gender was of 70.70% males and 29.30% females.

The individuals first signed an informed consent form, when they were educated about the aim of the tests and what the results would be used for. After the consent, they answered the anamnesis, from which we extracted their occupational past, including aspects such as noise exposure - duration and frequency, the association of other agents, risks for hearing impairment and clinical data. Among the anamnesis issues, there was one associated with the presence or absence of tinnitus which, if present, would direct the individual towards answering the Tinnitus Severity Questionnaire (TSQ)^14^.

The next stage was the clinical assessment, when we assessed the external acoustic meatus and did the threshold tonal audiometry, by air conduction in the frequencies of 500 Hz, 1, 2, 3 and 4 kHz (if the air conduction thresholds were equal to or greater then 25dB). The test procedure for both pathways was identical, following the ISO standard^19^ (1989), using audiometers calibrated according to international standards^20^ and respecting a hearing rest period of, at least, 14 hours. The audiograms were, then, classified according to the occupational criteria considered the most sensitive among many possible options^21^, ^22^, proposed by Merluzzi et al.^13^ (1979). Assuming that an important share of the hearing losses could be noise-induced (NIHL) in function of the series and occupational history of these people, the choice for the classification method still can be justified for being considered the most adequate to check the development of NIHL. Thus, added to the criteria in the anamnesis, we used the results from previous audiometry tests to measure and suggest the etiology for these hearing losses.

With the data taken from the hearing classification variable (Merluzzi groups from 0 to 5) we obtained the frequencies for each category, as well as for the level of tinnitus variable (from absent to catastrophic).

We considered as response variable the presence or absence of tinnitus, and as explanatory variables the Merluzzi groups. Considering the Merluzzi groups as ordinal category variables, we used the adjustment of a logistics model with accumulated logits.

Since the frequencies obtained considered the severity of tinnitus and the Merluzzi groups, we adjusted a generalized linear model for binomial data and checked the interaction between these two factors. We should also stress that the analyses were carried out considering the right and left ears separately and were carried out using the SAS software, version 8.0223.

We excluded categories Levels 6 and 7, because by definition, they did not have workers with hearing loss originating exclusively from occupational situations, also because only a handful of individuals in this study would fit these categories.

## RESULTS

The 284 workers available for the study made up a total of 568 ears. We took 13 right ears (4.58%) and 9 (3.17%) left ears off the study because they were classified in levels 6 or 7 of Merluzzi. Thus, we had 271 right ears and 275 left ears.

Table 2 shows information on the frequencies both for the hearing loss and for the tinnitus severity. Of the 275 left ears, in 106 (38.55%) there was no indication of a hearing impairment; while in 169 (61.45%) there was some degree of hearing loss; for 147 ears (53.45%), patients did not complain of tinnitus and 128 of them (46.55%) complained of it.

**Table 2:** Distribution of the individuals according to Merluzzi et al”s classification13 (1979) and to tinnitus severity according to McCombe et al.16 (2001), for both ears, in a cross-sectional study, Bauru, 2003.

		NT	NegT	MldT	ModT	ST	CT	Total
				Frequency (%)				
M0	LE	78 (28,36)	12 (4,36)	8 (2,91)	4 (1,45)	2 (0,73)	2 (0,73)	106 (38,55)
	RE	76 (28,04)	11 (4,06)	7 (2,58)	8 (2,95)	2 (0,74)	1 (0,37)	105 (38,75)
M1	LE	39 (14,18)	10 (3,64)	10 (3,64)	10 (3,64)	4 (1,45)	0 (0,00)	73 (26,55)
	RE	51 (18,82)	11 (4,06)	15 (5,54)	8 (2,95)	3 (1,11)	1 (0,37)	89 (32,84)
M2	LE	17 (6,18)	8 (2,91)	6 (2,18)	7 (2,55)	2 (0,73)	1 (0,36)	41 (14,91)
	RE	9 (3,32)	8 (2,95)	5 (1,85)	6 (2,21)	3 (1,11)	1 (0,37)	32 (11,81)
M3	LE	8 (2,91)	5 (1,82)	7 (2,55)	6 (2,18)	3 (1,09)	0 (0,00)	29 (10,55)
	RE	5 (1,85)	4 (1,48)	5 (1,85)	5 (1,85)	3 (1,11)	0 (0,00)	22 (8,12)
M4	LE	2 (0,73)	1 (0,36)	5 (1,82)	0 (0,00)	2 (0,73)	1 (0,36)	11 (4,00)
	RE	1 (0,37)	2 (0,74)	4 (1,48)	0 (0,00)	1 (0,37)	0 (0,00)	8 (2,95)
M5	LE	3 (1,09)	2 (0,73)	2 (0,73)	3 (1,09)	3 (1,09)	2 (0,73)	15 (5,45)
	RE	2 (0,74)	2 (0,74)	3 (1,11)	4 (1,48)	2 (0,74)	2 (0,74)	15 (5,54)
Total	LE	147 (53,45)	38 (13,82)	38 (13,82)	30 (10,91)	16 (5,82)	6 (2,18)	275 (100,0)
	RE	144 (53,14)	38 (14,02)	39 (14,39)	31 (11,44)	14 (5,17)	5 (1,85)	271 (100,0)

Legend:

NT - No Tinnitus

NegT - Negligible Tinnitus

MldT - Mild Tinnitus

ModT - Moderate Tinnitus

ST - Severe Tinnitus

CT - Catastrophic Tinnitus

M0 - Without hearing loss

M1 - M5 - NIHL levels from 1 to 5, respectively

For the right ears (271 ears), in 105 (38.75%) there was no indication of hearing loss, while in 166 (61.25%) there was some degree of hearing loss; for 144 ears (53.14%), individuals did not complain of tinnitus and for 127 of them (46.86%) there was some complaint of the symptom.

We have noticed that there is a certain gradient of tinnitus complaints. The proportion of individuals affected in relation to the total reduced in accordance to the increase in tinnitus severity, differently from what happens with hearing losses, because Table 2 shows that the number of individuals, for instance, within Merluzzi”s Level 5 (most severe) is higher when compared to those individuals with Merluzzi”s level 4 (less severe).

Table 3 shows that tinnitus prevalence within each of the Merluzzi”s group increases according to a worsening in the thresholds (from 27% at level 0 all the way to about 84% in levels 4 and 5), thus, since the chances of tinnitus occurrence (by means of odds ratio estimates, respective confidence intervals and statistical significances).

**Table 3:** Distribution of the individuals as to the prevalence of tinnitus in each Merluzzi group13 (1979) and estimates of tinnitus occurrence possibility, by stratum, in a univaried analysis, in a cross-sectional study, Bauru, 2003.

	Tinnitus prevalence (%)	OR	CI 95%	p-value
Merluzzi 0	27,0	1,00	–	–
Merluzzi 1	44,5	2,16	1,40-3,34	0,001
Merluzzi 2	64,4	4,88	2,77-8,61	<0,0001
Merluzzi 3	74,5	7,89	3,92-15,89	<0,0001
Merluzzi 4	84,2	14,41	4,04-51,31	<0,0001
Merluzzi 5	83,4	13,51	4,93-36,98	<0,0001

The odds ratio varies from 2.16 (in group 1) to more than 13 (in groups 4 and 5). To build the contingency tables and obtain these estimates, we considered Merluzzi”s Level 0 (normal hearing) as a reference level and as exposure factors for the other groups, always in univaried analysis, besides the very occurrence of tinnitus.

The motivation behind this study was to check if there is any relationship (or dose-response relationship) between the information provided by the audiometric findings indicating hearing loss and the tinnitus complaint, in other words, if the development of hearing loss is associated to an increase in the discomfort caused by tinnitus.

Table 4 shows the interaction tests among Merluzzi”s groups and tinnitus severity, considering as base curve the mildest levels of each one of the classifications, that is, the individuals who do not have hearing loss, nor complain of tinnitus. The grouped interaction (all hearing loss levels associated with all tinnitus levels) was statistically significant; however, when analyzed together (hearing loss level * tinnitus severity) alone, not all of them were significant. For the 12 pairs of left ears (57.15%) results were significant, whilst for the right ears, only 6 pairs (28.57%) presented p values ≤0.05. Despite this difference, the proportions-difference test was not statistically significant (Z=1.95, valor-p=0.051).

**Table 4:** Analysis of maximum true similarity estimates of the association between NIHL and tinnitus for both ears, in a cross-sectional study, Bauru, 2003.

Parameter	Estimate		Standard error		x^2^		p-value	
	OE	OD	OE	OD	OE	OD	OE	OD
Interceptor	0,97	1,71	0,06	0,07	225,80	534,99	<,0001	<,0001
M1 * MldT	0,70	0,72	0,22	0,28	10,13	6,45	0,0015	0,0111
M1 * ModT	2,83	-2,70	1,29	0,60	4,80	20,39	0,0284	<,0001
M1 * ST	1,64	0,43	0,62	0,53	7,08	0,66	0,0078	0,4178
M1 * CT	0,63	0,86	0,28	0,51	5,10	2,78	0,0239	0,0957
M2 * MldT	0,47	0,46	0,21	0,26	5,15	3,14	0,0233	0,0765
M2 * ModT	1,09	-2,41	0,89	0,58	1,51	17,34	0,2197	<,0001
M2 * ST	1,64	0,69	0,62	0,56	7,08	1,56	0,0078	0,2121
M2 * CT	0,53	0,64	0,27	0,49	3,85	1,69	0,0497	0,1936
M3 * MldT	0,22	0,33	0,20	0,25	1,29	1,70	0,2563	0,1925
M3 * ModT	-0,10	-1,67	0,64	0,54	0,02	9,48	0,8774	0,0021
M3 * ST	0,60	0,39	0,46	0,51	1,72	0,56	0,1892	0,4537
M3 * CT	0,36	0,42	0,26	0,46	1,89	0,86	0,1691	0,3549
M4 * MldT	-0,08	-0,25	0,19	0,22	0,18	1,32	0,6724	0,2498
M4 * ModT	-0,10	0,73	0,64	0,62	0,02	1,40	0,8774	0,2375
M4 * ST	-0,48	-0,25	0,37	0,47	1,68	0,28	0,1955	0,5989
M4 * CT	-0,15	-0,27	0,24	0,40	0,40	0,44	0,5267	0,5049
M5 * MldT	-0,45	-0,28	0,18	0,22	6,62	1,70	0,0101	0,1921
M5 * ModT	-2,07	1,79	0,67	0,64	9,46	7,78	0,0021	0,0053
M5 * ST	-1,46	-0,42	0,37	0,47	15,77	0,80	<,0001	0,3709
M5 * CT	-0,51	-0,38	0,22	0,40	5,22	0,94	0,0224	0,3332
Grouped**	–	–	–	–	129,70	111,99	<,0001	<,0001

** degrees of freedom = 20

## DISCUSSION

The noise induced hearing loss is one of the most prevalent occupational disease in the world^24^, ^25^. Horg and Raymond^26^ (2003), in a study carried out in the USA, found NIHL in about 60% of the 575 workers of civil construction they studied, while Monley et al.^27^ (1996), collecting audiologic information from 89,500 subjects from the Australian population exposed to damaging levels of noise found a prevalence of 57.7% of subjects with hearing alterations suggesting noise-induced hearing loss.

NIHL prevalence is also high in developing countries, like Brazil. Andrade and Schochat28 (1988) assessed 7,043 workers exposed to noise in the city of São Paulo, and found prevalence rates between 30 and 55%, depending on their field of activity. Miranda et al.^29^ (1998), studied 7,925 workers from 44 different industries in the metropolitan region of Salvador, and found overall NIHL prevalence of 36%. Manubens^30^ (1994) found the disorder in 23% of 32,007 workers from 150 processing industries from 16 Brazilian states.

Besides industrial exposure, some studies involving noise-exposed workers in non-industrial environments were also found. Cordeiro et al.^31^ (1994) found NIHL prevalence around 45% among 292 drivers and collectors from public transportation vehicles from the city of Campinas, whilst Martins et al.^32^ (2002), in a study carried out in Bauru, found the disorder in 37% of the workers with the same professional characteristics. Also with those drivers and collectors, Corrêa Filho et al.^33^ (2002), found a NIHL prevalence of about 33%.

As far as tinnitus is concerned, epidemiologic data is even scarcer, especially when associated with specific disorders. Thus, it is very difficult to assess its social impact. Estimates point out that in the United Kingdom, from 35 to 45% of the people have already had some kind of tinnitus^34^, numbers which are similar to the ones found in the USA^35^. Among them, 8% have sleep disorders, 1% has severe disorders and 0.5% has severe impact in their daily lives^35^. It is known that severe tinnitus is considered the worst symptom that may affect human beings, being less important only when compared to untreatable intense pain and dizzinnes^36^.

Exposure to noise is responsible for the most common cause of tinnitus^37^, ^38^, reported by about 25% of the individuals exposed to it^6^, ^38^. In this series, tinnitus prevalence was of approximately 48%, much higher than the one reported in the literature. The fact that tinnitus is subjective and may vary according to the emotional or physical status of the individual^39^, together with the scarcity of epidemiologic data and the fact that it is a symptom - and not a disease, with the non-existence of objective measurement methods or proper experimental models, are the factors responsible for bringing some more difficulty to investigate it.

Given the scarcity of epidemiological data about both problems, to establish methodological proposals to check the association and the interaction between them becomes very restricted.

However, with the data obtained from the present investigation, the dose-response relationship between hearing loss and tinnitus was identified through a statistical model, considering the evolution of the hearing damage and the progression of the tinnitus severity. As we interpret the results, especially those presented on Tables 3 and 4, we can conclude that they are enough to show that in milder hearing losses, tinnitus is less prevalent and less severe; on the other hand, in higher losses, the chance of the patient developing tinnitus is also higher. Based on the results, we can infer that there is a trend towards having more severe tinnitus on their left ears, corroborated by the grouped interaction information - statistically significant shown on Table 4. The fact that not all combinations show statistical significance may be due to the reduced number of workers grouped in each pair, due to the stratifications. One fact that drew our attention was a trend towards this relationship between hearing loss and tinnitus is stronger in left ears, even if the difference in statistics does not show significant results. There is no formal explanation for this difference, given that it is usually reported in populations that present unilaterally predominant noise exposure - drivers, for instance, which was a category of professionals that represented less than 10% of our sample. We require more studies to check and see if this trend towards unilateralism is plausible or if there is any biologic marker associated with such finding.

One more aspect that speaks in favor of this interaction between the occurrences is explained on Table 3, which shows statistically significant increases of the possibility of workers having tinnitus, regardless of the degree of hearing loss when compared to those individuals who did not have hearing loss.

In regards of the study limitations, it is relevant to establish the causal nexus associated with the hearing loss, if only noise-induced or if associated with some other etiology (s). The criteria considered in the study were based on the anamneses and on the occupational and audiological background of the worker, which are very important aspects associated with clinical practice; however, given the very complexity of the topic, they were too much simplistic. Thus, such situation must be considered and better assessed in new approaches regarding this topic.

## CONCLUSION

Results suggest that there is statistical interaction between hearing loss and tinnitus, with the trend that, the higher the hearing loss, the greater the discomfort caused by tinnitus, according to the data structure and the statistical model selected for such evaluation. Such results corroborate the clinical findings of this association, even though the study is limited as to sample size and characteristics - which can partially explain that fact that we did not find significant results in all the pairs obtained for data stratifications.

Even having these sample drawbacks in the inferential evaluations, the odds ratio estimates and the interaction found suggest that the evaluation was properly carried out and thus, foster the development of further, better defined studies and with larger samples, as well as involving different populations, given that hearing loss and tinnitus are highly prevalent in the population, and such condition can facilitate the recruitment of subjects. Thus, we can see that the interaction of these factors can be seen in any condition or stage associated with them.
